# Coping with Childbirth: Brain Structural Associations of Personal Growth Initiative

**DOI:** 10.3389/fpsyg.2017.01829

**Published:** 2017-10-31

**Authors:** Judith Mangelsdorf

**Affiliations:** ^1^Department of Psychology, Freie Universität Berlin, Berlin, Germany; ^2^Max Planck Institute for Human Development, Berlin, Germany

**Keywords:** personal growth initiative, structural MRI, vmPFC, proactive coping, PTSD, childbirth, posttraumatic growth, postecstatic growth

## Abstract

Major life events require psychological adaptations and can be accompanied by brain structural and functional changes. The goal of the current study was to investigate the association of personal growth initiative (PGI) as a form of proactive coping strategy before childbirth, with gray matter volume after delivery. Childbirth is one of the few predictable major life events, which, while being one of the most positive experiences for many, is also accompanied by multidimensional stress for the mother. Previous research has shown that high stress is associated with reductions in gray matter volume in limbic cortices as well as the prefrontal cortex (PFC). We hypothesized that PGI before childbirth is positively related to gray matter volume after delivery, especially in the ventromedial PFC (vmPFC). In a prospective study, 22 first-time mothers answered questionnaires about their PGI level 1 month before birth (T1) and 1 month after delivery (T2). Four months after giving birth, a follow-up assessment was applied with 16 of these mothers (T3). Structural brain data were acquired at both postpartal measurement occasions. Voxel-based morphometry was used to correlate prenatal PGI levels with postpartal gray matter volume. Higher PGI levels before delivery were positively associated with larger gray matter volume in the vmPFC directly after childbirth. Previous structural neuroimaging research in the context of major life events focused primarily on pathological reactions to stress (e.g., post-traumatic stress disorder; PTSD). The current study gives initial indications that proactive coping may be positively associated with gray matter volume in the vmPFC, a brain region which shows volumetric reductions in PTSD patients.

## Introduction

Major life events are often accompanied by high stress (Price and van Stolk-Cooke, 2015). They can disrupt our assumptions about the world in a way that requires fundamental cognitive changes to accommodate these experiences (Cann et al., 2010). One of the most impactful events across the life course of a woman is giving birth to a child (Mangelsdorf and Eid, 2015). Generally, most women consider childbirth as a positive experience (Neuhaus et al., 2002). However, it is accompanied by intense multidimensional stress for the mother (e.g., physical pain, emotional arousal, and psychological distress; Lowe, 2002) that can sometimes result in PTSD (4.6–6.3%; Dekel et al., 2017).

There are four psychological reactions that individuals show as an outcome of being exposed to highly stressful experiences. First, some individuals react with resilience and show nearly no psychological impairment (McFarlane and Yehuda, 1996). They bounce back to their original level of psychological functioning. Second, some people are affected by the experience to a degree, but are able to recover. They experience psychological impairment as a consequence of high stress and core belief disruption, but after a while find their way back to their original level of psychological functioning (Bonanno, 2005). In a recent study, Infurna and Luthar (2016) questioned the distinction between resilience and recovery trajectories, since depending on the analytical approach, they found less different trajectories than Bonanno (2005) using the same data. Third, the most extensively studied reaction to trauma exposure is post-traumatic stress disorder (PTSD; e.g., Foa et al., 2000; Yehuda, 2002). Individuals suffering from PTSD experience severe psychological impairment as a consequence of highly stressful events and recover very slowly, or not at all. In a meta-analysis on PTSD and childbirth, the prevalence of PTSD ranged from 1.3 to 2.4% at 1–2 months and 0.9 to 4.6% at 3–12 months postpartum (Andersen et al., 2012). Thus, for some women, childbirth can be a highly challenging event, associated with the risk of traumatization and post-traumatic stress.

Finally, some individuals react to high stress and core belief disruption with cognitive changes that excel their original level of psychological functioning. They not only recover from high stress, but also – after a period of adaptation – show posttraumatic (Tedeschi and Calhoun, 1996) or postecstatic growth (Roepke, 2013). Tedeschi and Calhoun (1996) introduced the concept of posttraumatic growth as a possible outcome of exposure to trauma. They found that some individuals report psychological benefits including deepened relationships, higher appreciation of life, more personal strengths, a stronger sense of meaning and spirituality, and more openness toward new possibilities. For a long time, this unidirectional approach established the idea that suffering might be a prerequisite for growth. Roepke (2013) revised this assumption by investigating the psychological consequences of life events with positive emotional valence. She found that also positive emotional peak experiences can be a catalyst for beneficial developments, especially in the areas of relationships, self-esteem, meaning, and spirituality. Roepke (2013) framed this psychological reaction to major life events with positive valence post-ecstatic growth.

Most studies that investigate posttraumatic as well as postecstatic growth were based on measures that rely on the retrospective self-perception of change (e.g., Tedeschi and Calhoun, 1996, 2004; Roepke, 2013). This circumstance has been identified by influential researchers in the field as one of the most critical methodological aspects in growth research since it entangles genuine growth with cognitive illusions (Frazier et al., 2009; Tennen and Affleck, 2009; Jayawickreme and Blackie, 2014, 2016). Thus, a critical question is how to measure the outcomes of stressful life events with other approaches but self-reports.

Whether individuals react to a stressful experience with resilience, recovery, PTSD, or growth depends on multiple factors, which all influence psychological reactions to stress.

### Moderators of Stress

Various psychological, medical, and situational moderators that alter the stress level during labor, and consequently its psychological outcomes, have been investigated. De Schepper et al. (2016) examined obstetric, midwifery team care and personal risk factors for the development of PTSD after childbirth. They found that an external locus of control during labor as well as low socio-economic status were associated with higher PTSD scores, while spontaneous vaginal birth, the perception of the midwife being in control of the situation, and the possibility to ask questions were important preventive factors. In accordance with these findings, Cipolletta and Sperotto (2012) found in a qualitative study that further humanization in hospital settings (e.g., less machines in the room and a close relationships to the midwife) and the possibility of more personal agency improve women’s experiences during childbirth.

Moreover, active coping has been identified as a critical mediator of exposure to highly stressful events, neuroendocrine regulation, and development of psychopathology in general (Olff et al., 2005). It is associated with good adaptation to stress, and thus can prevent psychological disorder (North et al., 2001; Olff et al., 2005) and may foster positive outcomes of stressful experiences. Schwarzer and Knoll (2003) distinguish four coping strategies: reactive coping (alluding to harm or loss), anticipatory coping (pertaining to imminent threat in the near future), preventive coping (focusing on uncertain threat in the distant future), and proactive coping (involving upcoming challenges that are self-promoting). They highlight the critical role of proactive coping as the prototype of positive coping that does not require negative appraisal, threat, or loss, but includes all efforts to develop general resources that facilitate processes striving for personal growth (Schwarzer and Knoll, 2003). Following this definition, one important proactive coping strategy and positive resource that can help people to cope and function successfully facing adverse events is personal growth initiative (PGI; Robitschek, 1998; Robitschek et al., 2012), which we have addressed in this research.

Previous neuroimaging studies mainly focused on brain structural differences relating to negative (traumatic) life events and possible negative consequences, including mental disorders (see Smith, 2005; Karl et al., 2006; Kühn and Gallinat, 2013). In contrast, structural MR studies related to the above-mentioned protective buffering factors, such as proactive coping mechanisms to brain structure, are lacking. The current study investigated the association of PGI, as a skill set that supports proactive coping, with the brain structure of young mothers.

### Personal Growth Initiative

Critical life events such as childbirth pose a challenge to the individual. Schwarzer and Knoll (2003) distinguish the four different, above-mentioned coping strategies depending on two dimensions: (a) certainty of the event and (b) timing of the coping strategy. Reactive coping describes coping processes following the event. Meanwhile, anticipatory coping, preventive coping, and proactive coping are prospective coping strategies, which are built and used before the event takes place. The authors define proactive coping as all efforts that a person undertakes in order to build universal resources which promote the advancement of personal growth and the accomplishment of critical goals (Schwarzer and Knoll, 2003). In contrast to reactive coping, proactive coping summarizes coping strategies which are developed and used before a challenging event occurs and are independent from the valence of the event encountered.

Robitschek (1998) introduced the concept of PGI as a critical antecedent for coping effectively with life challenges. PGI can be defined as a developed skill set for self-improvement that includes cognitive and behavioral aspects (Robitschek et al., 2012; Meyers et al., 2015).

Personal growth initiative (PGI) is a multidimensional concept that encompasses four subdomains: readiness for change, planfulness, using resources, and intentional behavior (Robitschek et al., 2012). Individuals with high PGI levels strive for personal growth, set realistic goals for their change processes, ask for help, and actively work on themselves to realize their goals. Individuals who display high levels of PGI experience more emotional, social, and psychological well-being (Robitschek and Keyes, 2009) and are less likely to suffer from depression, anxiety, and emotional distress (Robitschek and Kashubeck, 1999).

The main assumption underlying the concept is that individuals have an active and intentional role in their personal change processes (Robitschek et al., 2012). The PGI concept is based on the premise that effective coping and positive development following psychological challenges are at least partially based on intentional and motivational aspects. In summary, positive development does not happen by chance, but can be the result of intentionally striving for self-improvement.

Robitschek et al. (2012) state that PGI is expected to prevent psychological distress by providing a mindset that facilitates effective handling of difficulties. They argue that individuals with high PGI are more likely to perceive stressful events as opportunities for growth instead of threats. PGI encompasses cognitive and behavioral skills that include the belief that one can change one’s circumstances, active planning, and goal-setting strategies directed toward attaining improvement (Robitschek et al., 2012). PGI enables individuals to assert some psychological control over their lives even under otherwise uncontrollable conditions. It may therefore represent a particularly adaptive mindset in adverse situations, which is conceptually similar to the construct of hope (Shorey et al., 2007; Blackie et al., 2015) and might therefore even go beyond the scope of a mere prospective coping strategy. Thus, possessing the skill set of PGI before stress exposure might prevent the development of high stress levels after major life events, and by this means buffer stress-related physical consequences.

It is important to distinguish psychological growth, as referred to in the concept of PGI, from growth concepts such as posttraumatic (Tedeschi and Calhoun, 1996, 2004) or postecstatic growth (Roepke, 2013). While PGI implies an ongoing process, thriving for self-improvement (Robitschek et al., 2012), posttraumatic and postecstatic growth can be defined as multidimensional psychological change processes caused by disruptive cognitive processes through major life events.

### Brain Structural Changes after Highly Stressful Life Events

Most research on brain structural consequences of major life events focused on traumatic experiences, especially in individuals suffering from PTSD. Traumatic experiences are extraordinarily stressful events, and thus the research concerned dealt with the effects of high stress on the brain. Basic knowledge about the brains’ response to stress comes from functional neuroimaging studies. These studies revealed that four brain areas are fundamentally involved in processing and regulating stressors in humans, namely hippocampus, prefrontal cortex (PFC), amygdala, and brainstem (cf., Dedovic et al., 2009).

Structural alterations in highly stressed populations (i.e., PTSD patients) are localized in similar areas: patients suffering from PTSD have smaller gray matter volume in cingulate, frontal, temporal, and limbic cortices (including the hippocampus and amygdala) compared to trauma-exposed and nontrauma-exposed healthy participants (Smith, 2005; Karl et al., 2006; Kühn and Gallinat, 2013). In a recent meta-analysis by Kühn and Gallinat (2013), four regions were found to show smaller volumes in PTSD patients compared to trauma-exposed controls: the anterior cingulate cortex, the ventromedial PFC (vmPFC), the hippocampus, and the temporal pole/temporal gyrus. Since most of these studies were cross-sectional, the question remains unanswered, if smaller gray matter volumes are an antecedent or consequence of PTSD.

Longitudinal data from animal studies depict various neurobiological effects of stress exposure on the function and structure of different brain regions such as the hippocampus and PFC (e.g., Magarinos and McEwen, 1995; McEwen and Morrison, 2013). Mediated among others by cortisol, stress exposure leads to cell atrophy and consequent decrease in brain volume in the affected regions (Magarinos and McEwen, 1995). Animal data indicate that volumetric differences observed in PTSD patients might reflect volume reductions due to psychopathological development following traumatic events.

In contrast to the literature focusing on traumatic events and highly stressed populations, few studies explored neuronal association of successful coping with stressful life events (e.g., Rabe et al., 2006). Rabe et al. (2006) investigated the relationship between posttraumatic growth and frontal brain asymmetry in survivors of motor vehicle accidents. They found that increased relative left frontal activation was positively associated with PTG. Meanwhile, no study has measured structural brain correlates of coping or resilience.

### Aims of the Current Research

Major life events often cause high stress for the affected individual, which in turn requires effective coping and adjustment (Mangelsdorf and Eid, 2015). One of the main challenges of research which investigates the outcomes of major life events is the unpredictability of many of these events. Giving birth is an exception in this regard, given that it is a relatively predictable life event associated with intensive multidimensional stress for the mother.

The present study investigates the association of PGI and brain structure in pregnant women transitioning into motherhood. The current investigation is the first study known to the authors that systematically investigated the association of PGI as a preventive coping strategy with gray matter volume.

Previous research on brain structural correlates of major life events has mainly focused on neural change relating to pathological development, such as PTSD (e.g., Smith, 2005; Karl et al., 2006; Kühn and Gallinat, 2013). In contrast, research into preventive factors that may buffer stress and counteract brain volume reductions is scarce. We hypothesized that prenatal proactive coping as a preventive factor for pathological reactions to stress is particularly associated with PFC volume. The PFC plays a critical role in the perception of controllability of stressful experiences (Salomons et al., 2007; Maier and Watkins, 2011; Maier and Seligman, 2016). Activation of the PFC enables top-down inhibitory control over limbic and brainstem responses to stressful situations (e.g., pain), while perceived controllability extenuates experienced stress (Maier and Watkins, 2011). Maier et al. (2006) exposed rats to uncontrollable shocks in a shuttle box escape task. These rats were not able to learn to escape shocks in a different situation that was controllable. Seligman and Maier (1967) termed this effect of inactiveness in the face of traumatic shock learned helplessness (Maier et al., 2006). Interestingly, Maier et al. (2006) added an experimental group that was also exposed to uncontrollable shock but received picrotoxin to activate vmPFC during experimental treatment. This group, even though previously exposed to high stress through inescapable shock, did not react with learned helplessness, but actively escaped the shock.

The prospective coping strategy PGI can be linked to the concept of controllability of stressful situations. Individuals with high levels of PGI should in theory have a stronger feeling of control over stressful situations. Hence, we hypothesized that prenatal PGI might be positively associated with brain volume in the PFC after childbirth.

## Materials and Methods

### Participants

The participating women were a subsample of a larger study focusing on cognitive and neural changes throughout pregnancy and childbirth (peripartum period). Women who participated in the original study were invited to take part in the psychological online assessment in addition to the on-site tests and MR scans. Only healthy pregnant women who had never been pregnant previously beyond 8 weeks were enrolled. None of the participants had a history of neurological or psychiatric conditions. The study was conducted according to the Declaration of Helsinki, with approval from the Ethics Committee of the German Society for Psychology and the ethics commission of the Max Planck Institute for Human Development. The initial sample of the present investigation consisted of 22 women (age: *M* = 28.09, *SD* = 3.15). One subject had not only an outlier PGI score exceeding the 75 percentile by 1.5 times the interquartile range but also a conspicuous response style. The person answered nearly all items of the provided questionnaires with the highest possible option, with nearly no variance between the different items and finished the online questionnaire in a very short time. Therefore, this subject was excluded from further analyses. The results of the full sample including the outlier are provided in the Supplementary Table 1 for comparison. The final sample that took part in the prenatal online assessment (T1) and in the postpartal MR scans consisted of 21 women (age: *M* = 28.19 years, *SD* = 3.19). Some women (*n* = 5) dropped out after the first MR scan and did not take part in the follow-up assessment.

### Design and Procedure

The present investigation was embedded in a longitudinal study assessing neural and cognitive change during the peripartum period. Within that study, women underwent cognitive and psychological assessment in the last weeks of pregnancy (T1). Structural imaging data were acquired from the same women about 1–2 months (T2) and about 4 months (T3) after childbirth. MR scans took place solely after delivery, due to medical concerns over scanning pregnant women. Participants who agreed to take part in the online-questionnaire assessment and underwent an MR scan in the first months after delivery were included in the present investigation. For these participants, PGI scores accessed before childbirth (T1) were correlated with postpartal gray matter volume (T2) in a whole-brain regression analysis. Additionally, PGI scores before childbirth were correlated with gray matter volume in the same area at T3. The online assessment at T1 was carried out about 20 days before delivery (*M* = 19.69, *SD* = 10.66). The imaging session at T2 was carried out 39 days after delivery (*M* = 38.68, *SD* = 13.67), while the imaging session with the reduced sample at T3 was carried out about 4 months after childbirth (*M* = 135.59, *SD* = 29.17).

### Questionnaires and Online Assessment

Participants who took part in the psychological assessment were contacted via email and provided with a link to the online questionnaire, which was hosted on the survey software site Qualtrix. All questionnaires were provided in German. For that we used a translation–retranslation approach.

#### Personal Growth Initiative – Personal Growth Initiative Scale-II (PGIS-II; Robitschek et al., 2012)

The PGIS-II is a multidimensional 16-item scale that measures the degree to which individuals actively show initiative to thrive for personal growth. It includes four subscales: readiness for change (e.g., “I can tell when I am ready to make specific changes in myself.”), planfulness (e.g., “I set realistic goals for what I want to change about myself.”), using resources (e.g., “I ask for help when I try to change myself.”), and intentional behavior (e.g., “I actively work to improve myself.”; Robitschek et al., 2012). Participants indicated to which extent they agree with each of the 16 statements (Likert’s scale ranging from 0 “disagree strongly” to 5 “agrees strongly”). With α = 0.91, the scale had a good internal consistency, which is comparable to the results of other studies (e.g., Thoen and Robitschek, 2013; α = 0.90 and 0.91). The stability coefficient of the PGIS-II between T1 and T2 was *r* = 0.64 and *r* = 0.74 between T1 and T3.

As described above, one outlier PGI score was excluded before further analysis due to extreme data in all questionnaires and PGI scores exceeding 75th percentile by more than 1.5 times the interquartile range. The results of all analyses including the outlier can be found in the Supplementary Table 1.

### MRI Data Acquisition

Magnetic resonance imaging (MRI) scans were acquired using a 3T Magnetom Tim Trio MRI scanner system (Siemens Medical Systems, Erlangen, Germany) using a 12-channel radiofrequency head coil. High-resolution anatomical images were collected using a T1-weighted 3D MPRAGE sequence (TR = 2500 ms, TE = 4.77 ms, TI = 1100 ms, acquisition matrix = 256 × 256 × 192, sagittal FOV = 256 mm, flip angle = 7°, voxel size = 1.0 mm × 1.0 mm × 1.0 mm).

### MRI Data Analysis

Anatomical data were processed by means of the VBM8 toolbox^1^ with default parameters by Gaser and the SPM8 software package^2^. The VBM8 preprocessing involves bias correction, tissue classification, and registration. The ‘non-linear only’ modulation was applied in order to preserve the volume of a particular tissue within a voxel by multiplying voxel values in the segmented images by the Jacobian determinants derived from the spatial normalization step. Images were smoothed with a full-width half-maximum kernel of 8 mm. Statistical analysis was carried out by means of whole-brain regression implemented in SPM8. Age and total intracranial volume were entered as covariates of no interest. The resulting maps were thresholded with *p* < 0.001 and a statistical extent threshold (*k* > 1000 voxels), correcting for non-stationary smoothness (Hayasaka and Nichols, 2004).

## Results

### Descriptive Statistics

**Table 1** displays the descriptive statistics of the different scales and the MR results at each time point. Further analyses focused on the PGI results of T1 in order to measure PGI as a preventive proactive coping strategy.

**Table 1:** Descriptive statistics.

PGIS-II T1		PGIS-II T2		PGIS-II T3		vmPFC T2		vmPFC T3	
				
*M*	*SD*	*M*	*SD*	*M*	*SD*	*M*	*SD*	*M*	*SD*
4.11	0.37	4.12	0.46	4.15	0.42	0.5036443116	0.0517414991	0.5063973605	0.0510023263


*PGIS-II, Personal Growth Initiative Scale-II, [1-6]; vmPFC, gray matter volume in the vmPFC.*

### MRI Results

**FIGURE 1:**
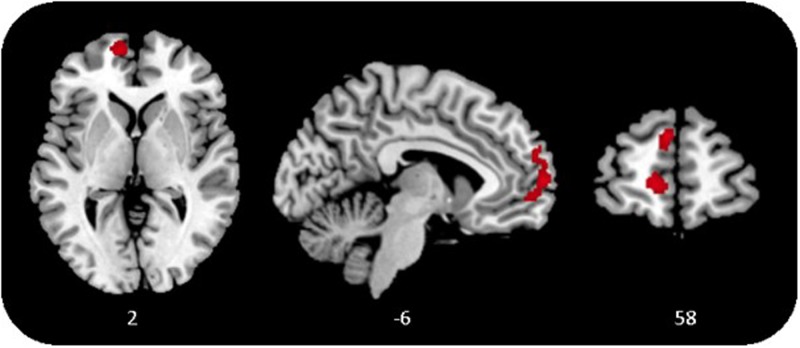
Gray matter in the ventromedial prefrontal cortex (vmPFC) correlates positively with personal growth initiative (PGI), *p* < 0.001, *k* > 1000 voxels, corrected for non-stationary smoothness.

Due to the small sample size and the occurrence of tied ranks, Kendall’s tau-b correlation coefficient (τ_b_; Howell, 2006) was used to estimate the association of prenatal PGI level and postpartal gray matter volume. A whole-brain voxel-based morphometry (VBM) regression analysis revealed a cluster in the left vmPFC with a significant positive correlation with PGI scores at T1 (τ_b_ = 0.38, *p* = 0.02; brain data acquired at T2; *p* < 0.001, *k* > 1000 voxels corrected for non-stationary smoothness; see **Figure 1**). No other regions were found to correlate positively or negatively with PGI. This effect also remained significant after Bonferroni correction.

In a confirmatory approach, the same vmPFC cluster, revealed in the whole-brain VBM regression with brain data acquired at T2, was used to estimate the association of PGI and vmPFC volume at T3. One-tailed Kendall’s tau-b correlation confirmed a positive relation of prenatal PGI and vmPFC volume, but failed to meet statistical significance (τ_b_ = 0.20, *p* = 0.13) in the reduced sample assessed 4 months after delivery.

## Discussion

The current study systematically investigated the relationship between prenatal PGI and postpartal brain structure. Mothers-to-be were provided with an online assessment approximately 1 month before birth, 1–2 months after delivery, and 3 months later. At the latter 2 time points, complementing MRI scans were realized. Individual differences in PGI scores were associated with gray matter volume in the vmPFC. Women with higher PGI scores before childbirth had larger vmPFC gray matter volume after delivery than those with lower PGI scores. The relation of prenatal PGI level and postpartal vmPFC volume was significant directly after delivery. However, it failed to meet significance at the follow-up assessment. This difference might be caused by participants dropping out, and consequently the diminished power of the analyses at T3. It is important to note that the correlational approach of this study does not allow assumptions about within-subject associations or causal relationships. However, the current findings give an initial indication of a positive connection between proactive coping and vmPFC volume, a brain region that is known to be impaired in individuals suffering from PTSD (Kühn and Gallinat, 2013).

The results allow different explanations, which will be addressed in more detail below.

Personal growth initiative (PGI), as a proactive coping strategy, might buffer the effects of exposure to high stress. This explanation is supported by psychological and neural evidence found in other studies. Proactive coping includes all efforts to build resources that promote successful mastering of future challenges and personal growth (Schwarzer and Knoll, 2003). Individuals with a high PGI level perceive potential stressors as opportunities for growth and cope with them by applying goal-directed and self-regulatory behaviors (Robitschek et al., 2012). This ensures a constructive path of action and increases the quality of functioning in the face of future challenges (Schwarzer and Knoll, 2003). Hence, individuals with higher PGI might be more resilient and experience less stress in critical and demanding situations, due to the resources and assets they have built.

The vmPFC is known to play a critical role in coping and resilience (Maier and Watkins, 2011). Studies on fear extinction in humans have found that the amygdala and vmPFC play a critical role in the acquisition and extinction of fear (e.g., Phelps et al., 2004; Milad and Quirk, 2012). While amygdala activation is associated with acquisition and early extinction of conditioned fear, the vmPFC is related to the long-lasting retention of extinction (Phelps et al., 2004). In their recently published article, Maier and Seligman (2016) introduced their revised theory of learned helplessness. They stated that passivity in response to inescapable prolonged stress is the default reaction to stressful events. This automated response is mediated by the serotonergic activation of the dorsal raphe nucleus (DRN), which can be turned off by the vmPFC when a stressor is detected as controllable. Maier and Seligman (2016) conclude that the key mechanism in response to inescapable stressors is not learned helplessness but learned controllability, which is detected and exerted by the vmPFC. Hence, proactive coping strategies, such as PGI, may lead to vmPFC activation in the face of stressful experiences by increasing perceived controllability. Since brain function can shape the brain structure within the same region (May, 2011), it is likely that this process also influences vmPFC volume. Maier and Seligman (2016) identified the presence of control confronted with stressors as “the active ingredient, leading to the inhibition of threat-induced changes in limbic and brainstem structures.” (*p*. 361). Since (chronic) high stress levels possibly cause structural changes (i.e., dendritic shrinkage) in the PFC (McEwen and Morrison, 2013), perceived control and resulting lower stress could mediate the association of PGI and vmPFC volume. Maier et al. (2006) found that preceding experiences with behavioral control over stress changes the vmPFC response to later stressors by also activating the vmPFC when the subsequent experience is uncontrollable. Maier and Watkins (2011) report that the experience of control over stressors alters the function of the vmPFC and associated brain regions: The changed activity of the vmPFC than inhibits stress-responsive structures and leads to stress resistance. Possibly, successful proactive coping that provides individuals with a sense of control over stressful experiences changes the vmPFC activation and consequently, in the long run, causes larger gray matter volume in this area. Hence, successfully coping with life challenges could influence their impact on the brain. From this explanation, PGI can be seen as a resilience factor, preventing stress and consequent neural losses.

An alternative explanation for the finding is that interpersonal differences in vmPFC gray matter volume are associated with cognitive and psychological functions that allow for a different extent of PGI. Yehuda et al. (2006) proposed that less gray matter volume could make individuals more vulnerable to the development of PTSD. The vmPFC plays an important role in counterfactual representations for future planning (Barbey et al., 2009). Hence, larger volumes might relate to differences in cognitive functions that are part of the PGI construct such as planfulness and intentional behavior. Participants who have greater PFC volume in the first place might – because of this biological asset – also have a greater capability for PGI. Following that explanation, PGI might not prevent individuals from losing brain volume under stress, but vmPFC volume might drive PGI levels, independently of the effects of stress.

### Personal Growth Initiative and Resilience

The stability coefficients of the PGIS-II, which were lower for the second measurement time point than for the third, suggest that the birth experience has a short-term influence on PGI level. The data indicate that some mothers show increased PGI scores directly after birth, while others react with a drop in the PGI level or maintained their PGI scores. These transitions can be explained by the challenging character of the childbirth experience and the resulting short-term effects on coping strategies. The majority of young mothers consider their delivery retrospectively as a very positive (65.5%) experience (Neuhaus et al., 2002). Meanwhile, in some cases, the birth process itself is connected to high negative stress for the mother and in the worst case might be experienced as traumatic. In a prospective study with pregnant women, about 5.6% of young mothers experienced acute postpartum trauma symptoms that met the DSM-IV criteria for PTSD (Creedy et al., 2000). The large range of possible birth experiences and following psychological reactions, such as resiliency, recovery, PTSD, or growth, might explain why PGI measured shortly after childbirth drops, increases, or maintains its former level. It is likely that women who experience recovery or PTSD react with impaired PGI, while resilient mothers or those who experience growth might show no changes or increased PGI scores. At the same time, it would not be likely that these short-term psychological changes in PGI are instantly accompanied by changes in brain volume. Therefore, only PGI scores assessed before childbirth were included in the analyses, which mirror proactive coping strategies and not reactions to stress exposure. Since we cannot test these explanations in the current sample, because of the small sample size and its cross-sectional MRI design, these associations should be investigated in future studies with larger samples that have the power to measure the impact of different childbirth experiences and its outcomes on proactive coping in the form of PGI.

### Limitations

#### Cross-sectional MRI Data

Even though the current study had a longitudinal design, ethical considerations prohibit scanning women during pregnancy. Therefore, it was not possible to acquire neural pre- and post-event MRI data (i.e., before and after childbirth). This methodological limitation prevents us from drawing definite conclusions about the causal relationship of the association between brain and PGI. Future longitudinal studies should aim to disentangle the association of PGI and vmPFC, as well as further investigate the association of both variables to the effects of stress.

#### Small Sample Size

An additional limitation of the study is its small sample size. Since childbirth is a highly stressful experience, emotionally and physically, for many women, recruitment for post-event MRI research is a challenge. However, since the described effects were found despite of the small sample size and the subsequent reduced power of the analyses, future studies should aim to replicate the findings.

#### Selectivity and Dropout

For this study, we recruited women who took part in the postpartum MRI scan. It can be assumed that this group is selective, since women who suffered from very high stress levels after birth or were longer hospitalized might not be included in this sample. The variance of the results might be limited by this fact. The additional dropout of five women might be responsible for the insignificant result at T3. Future studies should involve the hospitals in which the women give birth in order to reduce attrition rate and trace back systematic drop-out.

### Outlook

The current findings complement studies investigating how vulnerability and resilience after trauma exposure affect the brain. While the present study does not allow to draw causal conclusions, the possibility that PGI might be a buffer against stress caused by major life events and a source of gray matter changes in the vmPFC should be further explored. It might be possible that enhancing PGI before highly stressful life events, such as childbirth, enables individuals to cope more effectively, influences stress as well as vmPFC volume, and decreases the risk of developing PTSD symptoms. These possibilities must be further investigated in appropriately designed studies in order to draw final conclusions.

The effect of specific trainings that aim to promote PGI, such as the intentional growth training (Thoen and Robitschek, 2013), should be investigated in longitudinal neuroimaging studies. Thoen and Robitschek (2013) developed the Intentional Growth Training (IGT) with the goal to enhance PGI and thus enable cognitive and behavioral self-change, leading to better mental health. Future research should not only investigate the psychological benefits of such training but also systematically assess its effect on coping with high stress and its effect on the brain. PGI might be a valuable resiliency factor, especially for normative life events where preparation is possible.

While childbirth is a life event with high stress, it is also connected to various other hormonal, psychological, and physical changes which might also influence gray matter plasticity. Therefore, the association of PGI and gray matter volume after major life events should also be studied in other contexts (e.g., military deployment, natural disaster, or severe illnesses).

Finally, future research should explore the relationship of PGI and PTSD, since both might relate to the same neural structure. Assessing PGI, brain volume and PTSD prevalence in a longitudinal study design would allow the investigation of potential causal relationships between the three variables. This could help to discover preventive mechanisms for PTSD development and thus be of high clinical relevance.

## Conclusion

The current study set out to investigate how PGI and gray matter volume after childbirth are interrelated. PGI before birth was positively associated with postpartal gray matter volume in the vmPFC. This relation was significant in the larger sample assessed directly after delivery, and positive but not significant in the smaller sample 4 months later. Therefore, the current study should be seen as an initial indication and critical first step in broadening our understanding of neural correlates of proactive coping. Since a smaller volume of gray matter in this region is known to be related to high stress and PTSD, the finding suggests a new perspective on neural correlates of stress, focusing on coping and resilience. A broad body of research described the relation of small gray matter volume and potential neural losses after trauma exposure. Starting from our findings, future research should in addition consider the possibility of psychological and neural protection factors concerning major life events. The skill set of PGI is not specific to childbirth, but rather a universal tool. Hence, it can be presumed that it would also be beneficial in various other contexts. Investigating coping and protective psychological factors might not only inform research on PTSD prevention, but also unveil how to foster positive development across the life span.

## Author Contributions

JM developed the original research idea, collected the questionnaire data, and collaborated with the team of the MotherBrain study at the MPIB, Berlin, who provided the brain data. Additionally, JM performed the statistical analyses reported in the article except for the extraction of the brain data and the whole-brain regression analyses that were conducted by the MPI team. JM wrote and submitted the article.

## Conflict of Interest Statement

The author declares that the research was conducted in the absence of any commercial or financial relationships that could be construed as a potential conflict of interest.

## Footnotes

1 http://dbm.neuro.uni-jena.de/vbm.html

2 http://www.fil.ion.ucl.ac.uk/spm
