# Supplementary material for: Coping with Childbirth: Brain Structural Associations of Personal Growth Initiative
Source: Front Psychol. 2017 Oct 31;8:1829. doi: 10.3389/fpsyg.2017.01829 (PMC5671760; doi:10.3389/fpsyg.2017.01829)
Supplement: Supplementary file 1 [file Table_1.docx]

**Results including outlier (ID 1009)**

Supplementary Table 1

*Descriptive statistics including the outlier score*

| PGIS-II T1 | | vmPFC T2 | | vmPFC T3 | |
| --- | --- | --- | --- | --- | --- |
| *M* | *SD* | *M* | *SD* | *M* | *SD* |
| 4.18 | .49 | .5036391345 | .0504945386 | .5081007672 | .0500045201 |

*Note:* PGIS-II = Personal Growth Initiative Scale – II, [1-6]; vmPFC = gray matter volume in the vmPFC.

**MRI results**

Kendall’s tau-b correlation coefficient of

PGI at T1 and vmPFC volume at T2: τ_b_ = .35, *p* = .03

One-tailed Kendall’s tau-b correlation

coefficient of PGI at T1 and vmPFC volume at T3: τ_b_ = .22, *p* = .10
